# MinION Sequencing of Yeast Mock Communities To Assess the Effect of Databases and ITS-LSU Markers on the Reliability of Metabarcoding Analysis

**DOI:** 10.1128/spectrum.01052-22

**Published:** 2022-12-15

**Authors:** Angela Conti, Debora Casagrande Pierantoni, Vincent Robert, Laura Corte, Gianluigi Cardinali

**Affiliations:** a Department of Pharmaceutical Sciences, University of Perugia, Perugia, Italy; b Westerdjik Institute for Biodiversity, Utrecht, Netherlands; c CEMIN Excellence Research Centre, Perugia, Italy; The Hebrew University-Hadassah School of Dental Medicine

**Keywords:** MinION, Illumina, mock, species, yeast, LSU, ITS, metagenomic, database, delimitation, DNA sequencing, databases, metagenomics, yeasts

## Abstract

Microbial communities play key roles both for humans and the environment. They are involved in ecosystem functions, maintaining their stability, and provide important services, such as carbon cycle and nitrogen cycle. Acting both as symbionts and as pathogens, description of the structure and composition of these communities is important. Metabarcoding uses ribosomal DNA (rDNA) (eukaryotic) or rRNA gene (prokaryotic) sequences for identification of species present in a site and measuring their abundance. This procedure requires several technical steps that could be source of bias producing a distorted view of the real community composition. In this work, we took advantage of an innovative “long-read” next-generation sequencing (NGS) technology (MinION) amplifying the DNA spanning from the internal transcribed spacer (ITS) to large subunit (LSU) that can be read simultaneously in this platform, providing more information than “short-read” systems. The experimental system consisted of six fungal mock communities composed of species present at various relative amounts to mimic natural situations characterized by predominant and low-frequency species. The influence of the sequencing platform (MinION and Illumina MiSeq) and the effect of different reference databases and marker sequences on metagenomic identification of species were evaluated. The results showed that the ITS-based database provided more accurate species identification than LSU. Furthermore, a procedure based on a preliminary identification with standard reference databases followed by the production of custom databases, including only the best outputs of the first step, is proposed. This additional step improved the estimate of species proportion of the mock communities and reduced the number of ghost species not really present in the simulated communities.

**IMPORTANCE** Metagenomic analyses are fundamental in many research areas; therefore, improvement of methods and protocols for the description of microbial communities becomes more and more necessary. Long-read sequencing could be used for reducing biases due to the multicopy nature of rDNA sequences and short-read limitations. However, these novel technologies need to be assessed and standardized with controlled experiments, such as mock communities. The interest behind this work was to evaluate how long reads performed identification and quantification of species mixed in precise proportions and how the choice of database affects such analyses. Development of a pipeline that mitigates the effect of the barcoding sequences and the impact of the reference database on metagenomic analyses can help microbiome studies go one step further.

## INTRODUCTION

Metagenomics is defined as the direct molecular analysis of genomes, or parts of them, contained within environmental, agricultural, and clinical samples (1). It constitutes the most noteworthy event in the field of microbial ecology, because among other beneficial effects, it solves the problem of the viable noncultivable (VNC) microorganism (1, 2). Given the absence of full genomes of most organisms, metagenomics resorts to metabarcoding or amplicon-based metagenomics, which are the most widely used approaches for determining the microbial composition of a site (3, 4). Metabarcoding is based on next-generation sequencing (NGS) of marker genes: this usually involves regions of the rRNA gene (i.e., the 16S rRNA gene) for bacteria and the internal transcribed spacer (ITS), a sequence located between 18S and 26S rRNA in the rRNA precursor transcript, or large subunit (LSU), corresponding to the 26S rRNA gene, for fungi because these markers are ubiquitous and have hypervariable regions that differentiate species while being flanked by conserved regions that can be used to anchor “universal” primers (5). While the small subunit (SSU) marker is extremely useful at higher taxonomic levels, it has been proven to have insufficient resolution at the genus or species level (6).

The procedure for profiling microbial communities requires a number of technical steps that could produce a distorted view of the real community composition. Biases, in fact, can arise from sample collection and storage methods, DNA extraction, PCR amplification, DNA sequencing, bioinformatics, and statistical analyses. For this reason, mock communities have been used to evaluate the performance of a process (7, 8). For instance, Hallmaier-Wacker and colleagues examined a mock community composed of 22 bacterial strains and found that the choice of storage buffer and extraction kit affects the detected bacterial composition (9). Likewise, O’Sullivan et al. explored the impact of the bioinformatic approaches on microbiome assessment using 16S rRNA gene sequencing results generated from two mock microbial communities (10). In order to achieve accurate sequencing results, many factors have to be considered when designing a sequencing study. Among the processing steps that affects metagenomic analyses, PCR-based strategies are source of biases because of differential amplification efficiency among templates in terms of target length, primer binding sites and GC content (11–13). Many studies demonstrate potential amplification biases that are introduced with the use of various commonly utilized primers. Fouhy et al. demonstrated that the use of different primer sequences for the 16S rRNA gene (V4-V5, V1-V2, and V1-V2 degenerate primers) produces variable community profiles that differ both from the expected results and when comparing results obtained with the three primer sets. All of the primer sets detected false hits, which were present at low relative abundances and were closely related to the actual species present in the mock communities, suggesting misassignment at the species level due to similarities in the 16S rRNA gene sequence (14). Similarly, Bellemain and colleagues documented how the most commonly used fungal ITS primers were hampered by different types of biases: length bias, taxonomic bias, and primer mismatch bias (15).

To minimize the effect of such biases, various authors have suggested the use of different primer combinations so that different ITS regions would be analyzed in parallel. Various primers are used to amplify parts of the ITS region because the entire ITS region is too long for 454 sequencing or other high-throughput sequencing methods. The regions ITS1 and ITS2 provide greater taxonomic and functional resolution and richness of operational taxonomic units (OTUs) at the 97% similarity threshold compared to barcodes located within the ribosomal small subunit (SSU) and large subunit (LSU) genes (16) and were therefore chosen as the universal fungal markers (6). On the contrary, Mota-Gutierrez and colleagues registered cases of both underestimations and overestimation of species considering marker sequences of ITS2, due to the uneven length of such fragments. Furthermore, they demonstrated that the LSU region provided a higher α diversity index and greater fungal rRNA taxonomic depth and robustness results than ITS2 (17). Third-generation sequencers, and in particular Nanopore technologies, were built to produce long reads to overcome length limitations and provide a full-length sequence of ribosomal DNA (rDNA) cistron for improving identifications at the species level (18, 19).

Despite all these limitations, amplicon-based metabarcoding remains pivotal for environmental microbiology. The question is whether to consider the relative presence of a species as a sort of semiqualitative piece of information or if it can be considered quantitatively too. In general, the abundance of rDNA sequences from different microbes is used as an indirect measure of the abundance of the microbial taxa in the community, considering that the proportion of reads assigned to each group reflects the relative abundance of putative taxa within the sample. However, this assumption does not take into account intrinsic limitations of these markers, such as their multicopy nature, which is estimated to vary from 14 to 1,442 copies in fungi (20). Variation in genomic rDNA copy number could affect the measure of the relative abundance, because species with relatively low copies of the rRNA operon would be underestimated, whereas those with more copies would be overestimated. Lavrinienko pointed out the limits of using NGS-based methods to accurately quantify the taxonomic composition of eukaryotic microbial communities due to interspecific and intraspecific variations in the rRNA locus, emphasizing that copy number variation may confound analyses of microbial community composition. Thus, they suggested the need to adjust the counts of reads assigned to a particular taxon with taxon-specific values of rDNA copy number per genome (21). Whether for eukaryotes, this would be challenging, Kembel and colleagues presented a method that allows estimation of organismal 16S rRNA gene copy number and abundance by using ancestral state reconstruction via phylogenetically independent contrasts (22). In contrast, Starke et al. provided empirical evidence that gene copy normalization does not improve the 16S rRNA gene target sequencing analyses in real scenarios (23). Another fundamental step in metagenomic analysis is the choice of reference databases. It has been demonstrated that curated and smaller databases performed more precise predictions. In fact, the presence of more sequences in a given database increases the probability of genera being identified as a different taxon. In a previous paper, we proposed a pipeline to optimize the mapping against a reference with a two-step procedure based on the determination of the most likely species that were introduced in a dedicated smaller reference database for the final precise identification (24).

The aim of the present work is the evaluation of both the impact of different databases (the UNITE General Reference [GR] database and CBS reference database) and barcoding markers on metabarcoding studies carried out with long reads. For this purpose, we compared the relative abundances obtained with the analysis of Oxford Nanopore sequences and those sequenced with Illumina MiSeq. Six fungal mock communities were created in order to compare the expected and observed results. Furthermore, a two-step procedure, consisting of a preliminary identification followed by the definition of custom reference databases to carry out the second step, was proposed to increase the accuracy of metabarcoding analysis.

## RESULTS

### Assessment of species abundance using MinION sequences.

Long-read metabarcoding is a novel technique that could increase the possibility of accurate identification of both single species and environmental samples. Whereas Illumina sequences for taxonomic metabarcoding normally cover only the ITS2 sequence, MinION can span the entire DNA region, including ITS and LSU D1/D2, thus increasing the amount of information (ca. 1,200 bp versus 400 bp) and therefore the taxonomic resolution (25). Mock communities were generated to assess the applicability of third-generation sequences for a comprehensive description of simulated microbiomes composed by uneven proportions of the different species, as happens in real situations.

Each mock community mixture was obtained by mixing amounts of genomic DNA proportional to the chosen proportion of species abundance. This strategy was chosen rather than that of mixing known amounts of cell of the different species to avoid the bias derived from differential extraction efficiency. Two independent replicas of every mock community were carried out to assess the differences due to the whole series of molecular and bioinformatic operations before the species attribution step. The effects of different reference databases and molecular markers were evaluated.

**FIG 1:**
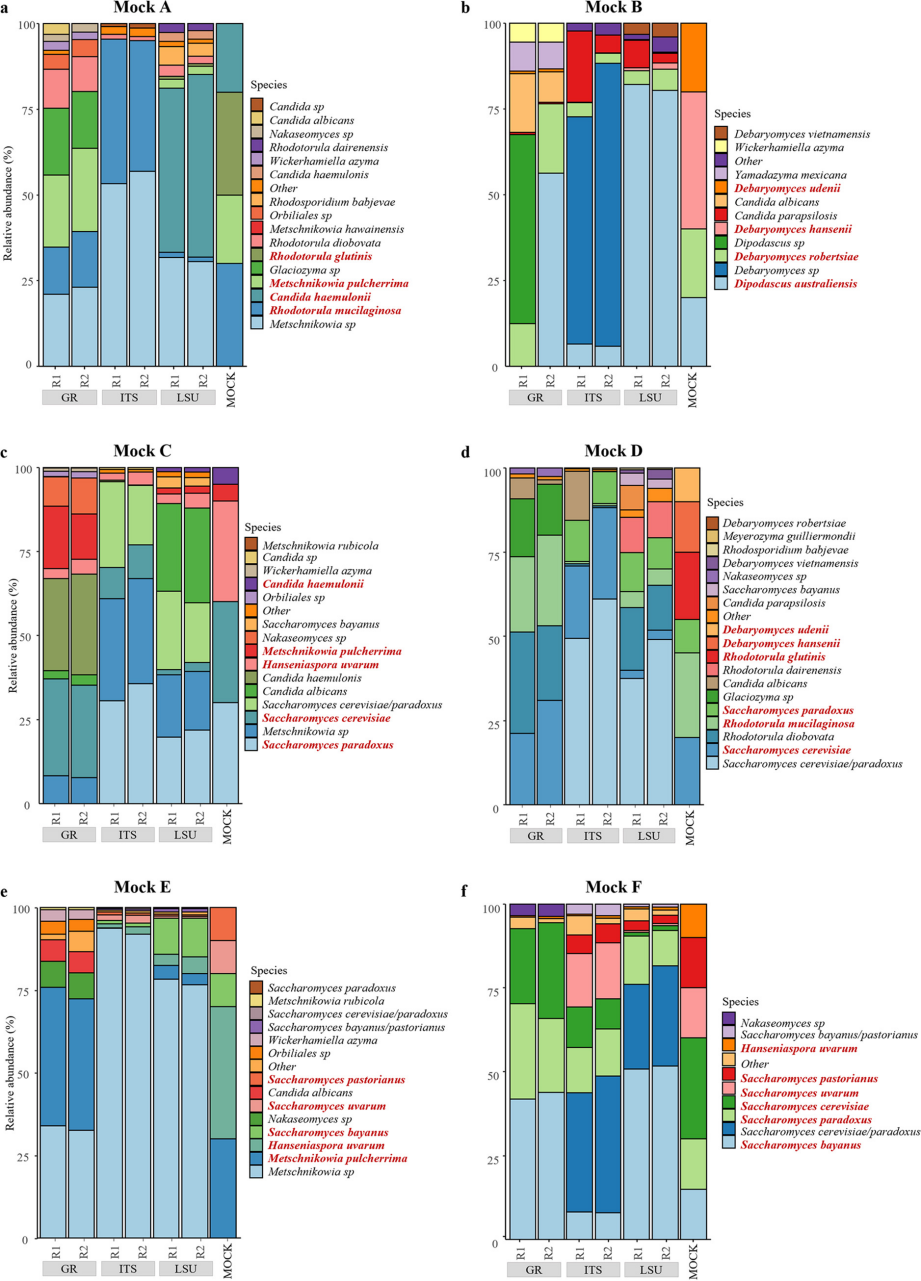
Fungal diversity of mock communities considering full databases (GR). Each panel shows the abundance (*y* axis) of species found within a mock community, obtained considering three different databases (*x* axis) using MinIon sequences. GR is the database composed of ITS sequences taken from UNITE, while ITS and LSU comprise sequences from CBS database. For each reference database, there are two columns that represent the two biological replicates (labeled R1 and R2). The right-most column of each panel represents the supposed abundance. Species present in the mock community are written in red, while the species in black are those identified by the mapping without being added initially. Mock community A contains 30% *R. glutinis*, 30% *R. mucilaginosa*, 20% *C. haemulonii*, and 20% *M. pulcherrima*. Mock community B contains 40% *D. hansenii*, 20% *D. robertsiae*, 20% *D. udenii*, and 20% *D. australiensis*. Mock community C contains 30% *H. uvarum*, 30% S. cerevisiae, 30% S. paradoxus, 5% *C. haemulonii*, and 5% *M. pulcherrima*. Mock community D contains 25% *R. mucilaginosa*, 20% *R. glutinis*, 20% S. cerevisiae, 10% S. paradoxus, 15% *D. hansenii*, and 10% *D. udenii*. Mock community E contains 40% *H. uvarum*, 30% *M. pulcherrima*, 10% S. bayanus, 10% S. pastorianus, and 10% S. uvarum. Mock community F contains 30% S. cerevisiae, 15% S. bayanus, 15% S. pastorianus, 15% S. uvarum, 15% S. paradoxus, and 10% *H. uvarum*.

**(i) Effect of the full reference database.** The influence of the reference database on defining the relative abundance of the single species or taxa is a methodologically relevant question in amplicon-based metagenomics. In a previous paper (26), it was already demonstrated that Illumina NGS outputs from single-strain sequencing of the ITS-LSU marker region produced lower homology values than expected from analogous Sanger sequencing, when using large databases. Subsequent reidentification with a smaller reference database, containing only the putative taxa obtained from the first alignment, produced homology percentages like those obtained with Sanger sequencing. This observation raised the question of the effect of reference database composition and richness on attributing NGS reads to known species, especially in the very complex context of amplicon-based metagenomics. In this work, three reference databases were tested, with six different mock community mixtures, for the taxonomical identification of long reads obtained with the MinIon sequencing platform. One is the “General Reference database” (herein referred to as UNITE, GR, or full reference database), obtained from the UNITE database and composed of 58,440 ITS sequences. The other two (CBS-ITS and CBS-LSU) were derived from the CBS database, containing 34,683 ITSs and LSU D1/D2 obtained from the Westerdijk Fungal Biodiversity Institute. The relative abundances obtained with the three databases in each of the six mock communities were compared with the expected species abundances of each mock community (Fig. 1).

From a general overview of the abundances, all three databases were found to have introduced many nonpresent species (hereinafter referred to as “ghost species”) in the original mock communities. When ghosts were members of the same genus, the erroneous identification could be ascribed to a lack of the necessary taxonomic resolution.

It could be noted that the species of the genus *Metschnikowia* were overestimated by all three reference databases in all of the mock communities where such species were present (Fig. 1a, c, and e). The CBS database had higher proportions of *Metschnikowia*, while UNITE found a relevant percentage of the ghost *Glaciozyma* species (Fig. 1a). Rhodotorula mucilaginosa was identified by all three databases, but it was underestimated by the UNITE (Fig. 1a and d) and CBS-LSU databases, while it was overrepresented in the CBS-ITS database (Fig. 1a). On the contrary, Rhodotorula glutinis was never detected but there was an excess of the ghost species Rhodotorula diobovata (Fig. 1a and d), leading to the conclusion that there was a misidentification due to the phylogenetic similarity of the two species (similarity of 0.979). Species of the genus *Dipodascus* were overestimated by the UNITE and CBS-LSU databases (Fig. 1b). The CBS-ITS database identified 75% of *Debaryomyces* spp. in mock community B (Fig. 1b), composed of four different species of *Debaryomyces* (80%) and one species of *Dipodascus* (20%), implying that the estimate at the genus level was almost correct without indications at the species level. Debaromyces robertsiae was the only species correctly identified by all three databases but with a strong underestimation. In general, *Debaryomyces* species were misclassified by both the UNITE and CBS databases (Fig. 1b and d), similarly to Hanseniaspora uvarum, which was strongly underestimated in all mock communities in which it was included, even in high abundance, as, e.g., in mock community E, where it represented 40% of the species (Fig. 1e). Mock communities, including some phylogenetically close species of the *Saccharomyces* genus, showed a strong underestimation of Saccharomyces pastorianus and Saccharomyces uvarum by the UNITE database and a high overestimation of S. bayanus (Fig. 1f) or of *Nakaseomyces* sp. (Fig. 1e). A similar trend was found when considering CBS-LSU in the same mock communitiess, indicating a scarce difference of efficacy of the two markers. On the other hand, CBS-ITS detected a lower percentage of S. bayanus, while it tended to classify both S. cerevisiae and S. paradoxus as “Saccharomyces cerevisiae/Saccharomyces paradoxus*”* hybrids. While S. cerevisiae was well estimated by UNITE, S. paradoxus was estimated at 22% versus the expected 15%.

**FIG 2:**
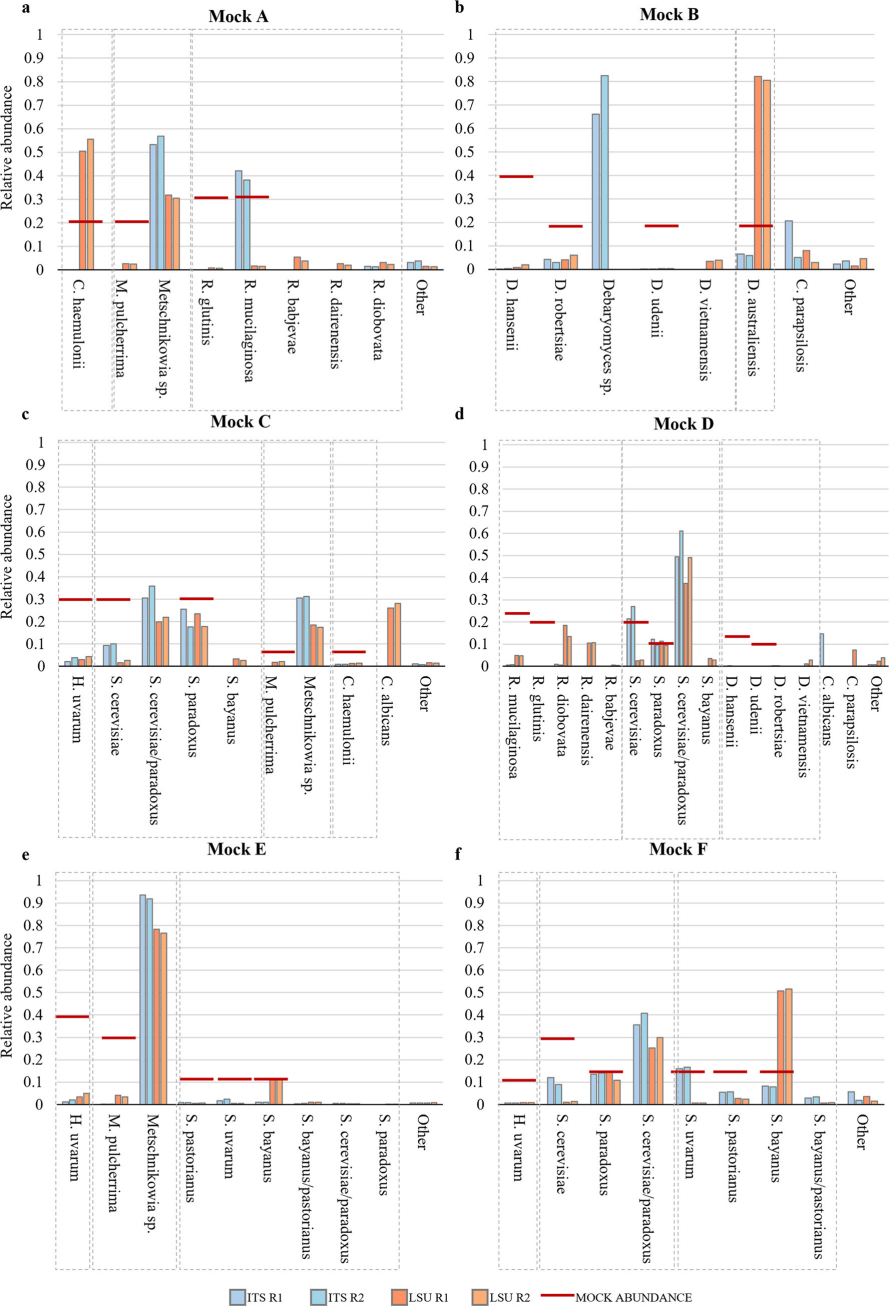
Different abundances obtained from different markers. The bar plots depict the relative abundance (*y* axis) of each species within the simulated communities, calculated considering separately ITS and LSU. The *x* axis shows all of the species identified in a mock community, grouped by genera. For each species, there are four bars representing the two replicas for each marker. The light blue bars indicate the relative abundance calculated with the ITS, while orange bars represent the abundances of species considering the LSU marker. Red horizontal lines show the expected value of abundance for each species, which were combined to create the simulated community: thus, species in the *x* axis that do not display the red line are considered ghost species (detected but not actually included).

**(ii) Difference in performance of ITS- versus LSU-based reference databases.** From the analysis of the relative abundances, carried out considering both CBS-ITS and CBS-LSU databases, different performance of the two markers was shown, with cases of oppositely erroneous estimates: i.e., one would overestimate and the other underestimate the expected relative abundance of the species. This suggests that the simultaneous use of both markers could produce a better estimate of the real proportion of the species. For example, Dipodascus australianensis and Candida haemulonii were identified at the species level, but LSU overestimated and ITS underestimated the expected abundances (Fig. 2a and b).

On the contrary, both ITS and LSU did not identify Metschnikowia pulcherrima correctly at the species level, but detected it as *Metschnikowia* sp., missing the species-specific level (Fig. 2a and e). The two species *Rhodotorula glutinis* and *R. mucilaginosa* were misclassified as four species of this genus with CBS-LSU in all mock communities (Fig. 2a and d). Conversely, the mapping with CBS-ITS returned a quite good estimation of *R. mucillaginosa* (Fig. 2a). The genus *Debaryomyces* showed a trend like *Metschnikowia*; in fact, it was not correctly classified at species level, but the abundance of *Debaromyces* sp. can be compared the expected abundance of the species of this genus in the mock communities. As in *Rodhotorula*, CBS-LSU distributed the species abundances among other species of the same genus, leading to the presence of some ghosts (Fig. 2b and d). Hanseniaspora uvarum was always severely underestimated with all reference databases in all mock communities. Saccharomyces cerevisiae and Saccharomyces paradoxus were relatively well quantified by CBS-ITS database, with some uncertainty, which was mostly due to the phylogenetic similarity between these species and the presence in the database of the hybrid species “S. cerevisiae*/paradoxus”* (Fig. 2c, d, and f). Saccharomyces bayanus was always overrepresented by CBS-LSU in the mock communities, where it was included and was detected as a ghost species in mock communities C and D, in which it was not included (Fig. 2c and d). Saccharomyces pastorianus and Saccharomyces uvarum were almost never identified by CBS-LSU, while CBS-ITS gave a relatively faithful representation of S. uvarum abundances in the various mock communities in which it was present.

### Comparison of MinION and Illumina.

Given the short length of the reads generated with the Illumina platform, ITS2 is largely used in fungal amplicon-based metabarcoding, rather than the whole ITS that was designed as a universal marker. For this reason, we compared ITS sequencing data obtained from MinION sequences with the corresponding sequencing data from the ITS2 region obtained with Illumina MiSeq. In both cases, UNITE was used as reference database for sequence mapping. The data shown in the two paragraphs below describe the behavior of the ITS from MinION and ITS2 from Illumina at the genus and species levels, respectively.

**(i) Performance of full reference databases at the genus level.** The first evidence that emerged from the analysis at the genus level of all mock communities is the decrease in ghost species with Illumina compared to the corresponding results obtained with MinION (see Fig. S1 in the supplemental material).

As a trade-off, species of the genera *Metschnikowia* and *Dipodascus* were never detected by Illumina (Fig. S1a, b, c, and e), while they were overestimated by MinION, suggesting that the former platform is less sensitive than the latter. On the contrary, the *Rhodotorula* genus was overrepresented by Illumina and underrepresented by MinION in mock communities A and D (Fig. S1d). The genus *Saccharomyces* was well estimated by both platforms, although mock community E showed little overrepresentation of this genus when Illumina was used. On the other hand, MinION did not recognize the *Saccharomyces* genus in mock community E, but it detected several ghost species. *Hanseniaspora* was always severely underestimated by MinION but well identified by Illumina. In order to compare the performance of the two platforms in the different mocks, two scores were developed to evaluate the matching of estimated and observed values from both qualitative (matching index 1 [MI-1]) and quantitative (MI-2) viewpoints. In the former score, the factors refer to the presence/absence of the expected versus observed pairs, irrespective of the actual value of the estimate. All of the three possible cases, true positives (TPs), false negatives (FNs), and false positives (FPs), are given a score of 1 and introduced in equation 1. The quantitative MI-2 index accounts for the mismatch between the estimated and observed percentages as described in Materials and Methods.

According to both MI-1 and MI-2, Illumina outperformed MinION at the genus level; in fact, the average MI-1 scores were 0.4 and 0.36, whereas the MI-2 scores were 0.63 and 0.39, respectively, for Illumina and MinION, indicating that the there was a strong quantitative difference, whereas the discrepancy at the qualitative level was not particularly high (Fig. S2).

**FIG 3:**
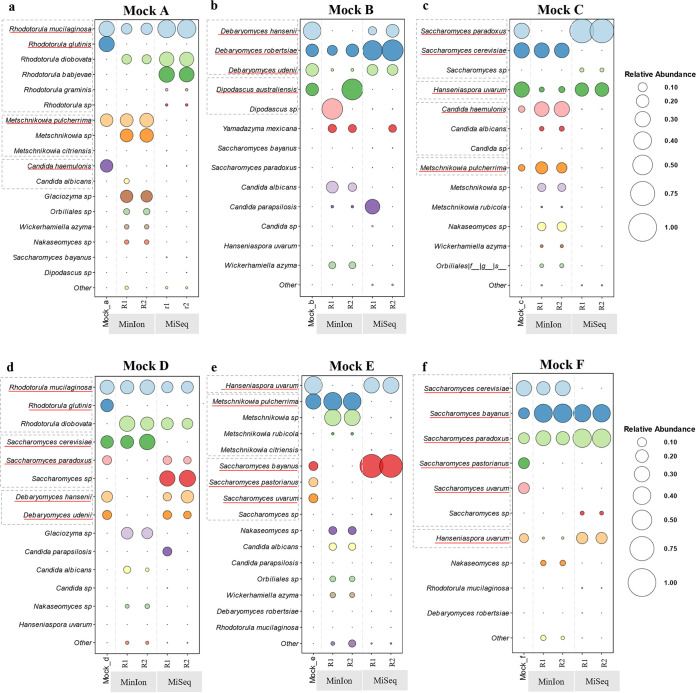
Comparison of fungal diversity of mock communities at the species level obtained with MinION and Illumina MiSeq, considering the full reference databases (ITS). Each panel shows the abundance of species found within a mock community, expressed with circles of different magnitudes that increase proportionally with the increase of relative abundance from 0 to 1. The database used for the mapping is composed only of ITS sequences in order to compare the two different sequencing methods. For each mock community, the results are summarized in five columns. The left-most column of each panel represents the supposed abundance present in the mock community, and the following two columns represent the two biological replicates (labeled R1 and R2) obtained by MinION, while the last two are those obtained by MiSeq.

**(ii) Performance of the full reference databases at the species level.** According to the results at the species level, Illumina did not precisely identify the species present but identifications tended to be distributed among different species within the same genus, even if they were not all present (Fig. 3).

**FIG 4:**
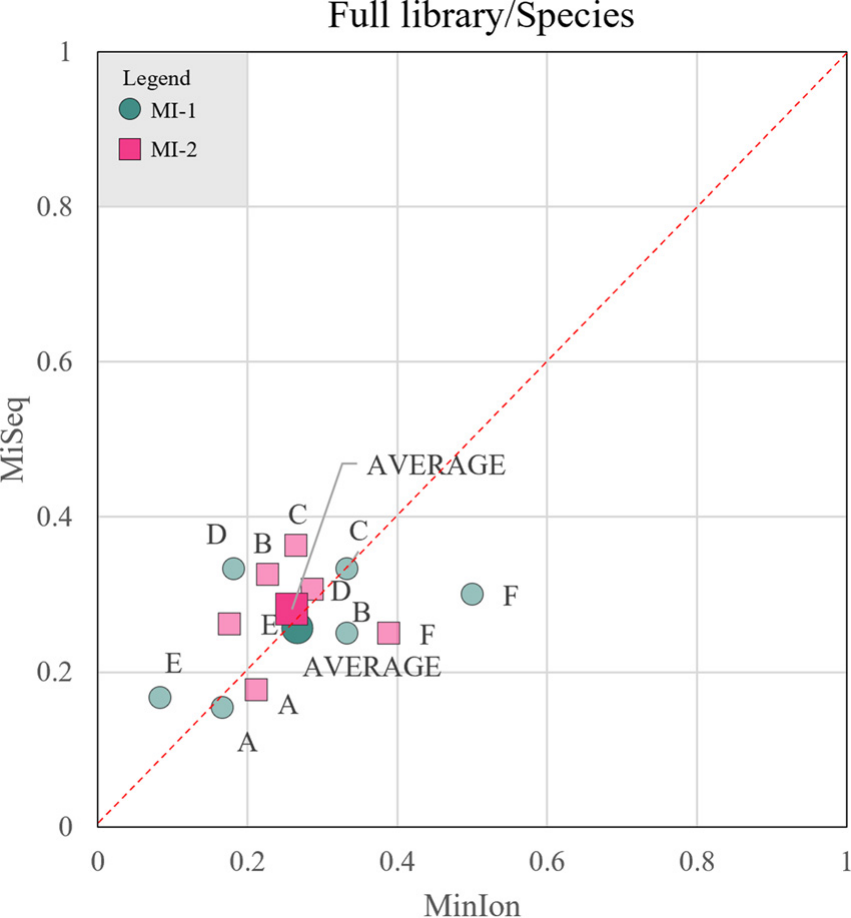
Comparison of the performance of MinION and MiSeq at the species level by a qualitative and a quantitative approach, considering a full ITS reference database. In order to compare the performance of the two sequencing platforms, two scores were developed to evaluate the matching of estimated and observed values from both a qualitative (MI-1 [pink squares]) and quantitative (MI-2 [dark blue-green circles]) viewpoint. Each mock community (labeled A, B, C, D, E, or F) is characterized by the two scores calculated at the species level for both MinION (*x* axis) and MiSeq (*y* axis), considering a full ITS database. The average value for each score, obtained considering all mock communities, is characterized by a bigger marker.

For example, in mock community A, Illumina detected five different species of *Rhodotorula* instead of the only two present; on the contrary, MinION recognized one of the two and wrongly identified the second, underestimating the expected abundance (Fig. 3a). In general, Illumina underestimated and MinION overestimated the number of species present, introducing ghost species. Evidence of this phenomenon is shown in mock communities A and C, where *M. pulcherrima* and *C. haemulonii* were not identified by Illumina. Conversely, MinION detected 4 species absent in the original mixture (Fig. 3a and c). Illumina precisely estimated the percentage of *H. uvarum* and correctly classified *Debaryomyces* species, while MinION severely underestimated both of them (Fig. 3b to d and f). A critical issue that arose with Illumina sequencing is that species of the *Saccharomyces sensu stricto* group could not be distinguished (Fig. 3c, e, and f) due to the close phylogenetic relationship. Only S. bayanus and S. paradoxus were identified, while MinION could differentiate the latter from S. cerevisiae. Using the matching indices described above, the MI-1 averages for Illumina and MinION were 0.26 and 0.27, respectively, while the MI-2 outputs were 0.28 and 0.26, respectively, indicating that using full reference databases, both platforms had similar and poor outcomes (Fig. 4).

With a mean of 1,200 bp, MinION sequences were longer than the query sequences of UNITE, averaging around 506 bp (Fig. S3 and Table S4), implying a possible bias due to the different sequence lengths. In fact, the ghost sequences introduced by MinION were mostly *Orbiliales* sp., *Glaciozyma* sp., and *Nakaseomyces* sp., whose respective lengths are 1,390, 1,193, and 1,360 bp, confirming that the mapping procedure tends to identify species with longer sequences in the reference database.

Furthermore, a phylogenetic analysis of the species involved in the study demonstrated the presence of a relationship between the aforementioned ghost species and the species used for the mock communities (Fig. S4), indicating that these problems arise from a combination of the different lengths of this marker and the phylogenetic similarity.

### Use of specific databases to mitigate the species abundance problems.

Since full databases with many species may result in abundance estimations far from the proportion of each mock community, a simple two-step pipeline was conceived consisting of a preliminary identification followed by the definition of a “dedicated database” to carry out the second step. The “dedicated database” is restricted only to the strains (and maybe only to the type strains) of all the species found in the first identifications, and it is used to carry out the second identification. The analysis at the genus level carried out with this approach did not produce significantly great improvements according to the MI-1 and MI-2 scores (Fig. S5) and led us to concentrate on the species level, which is the preferential target of many studies on fungal communities.

**FIG 5:**
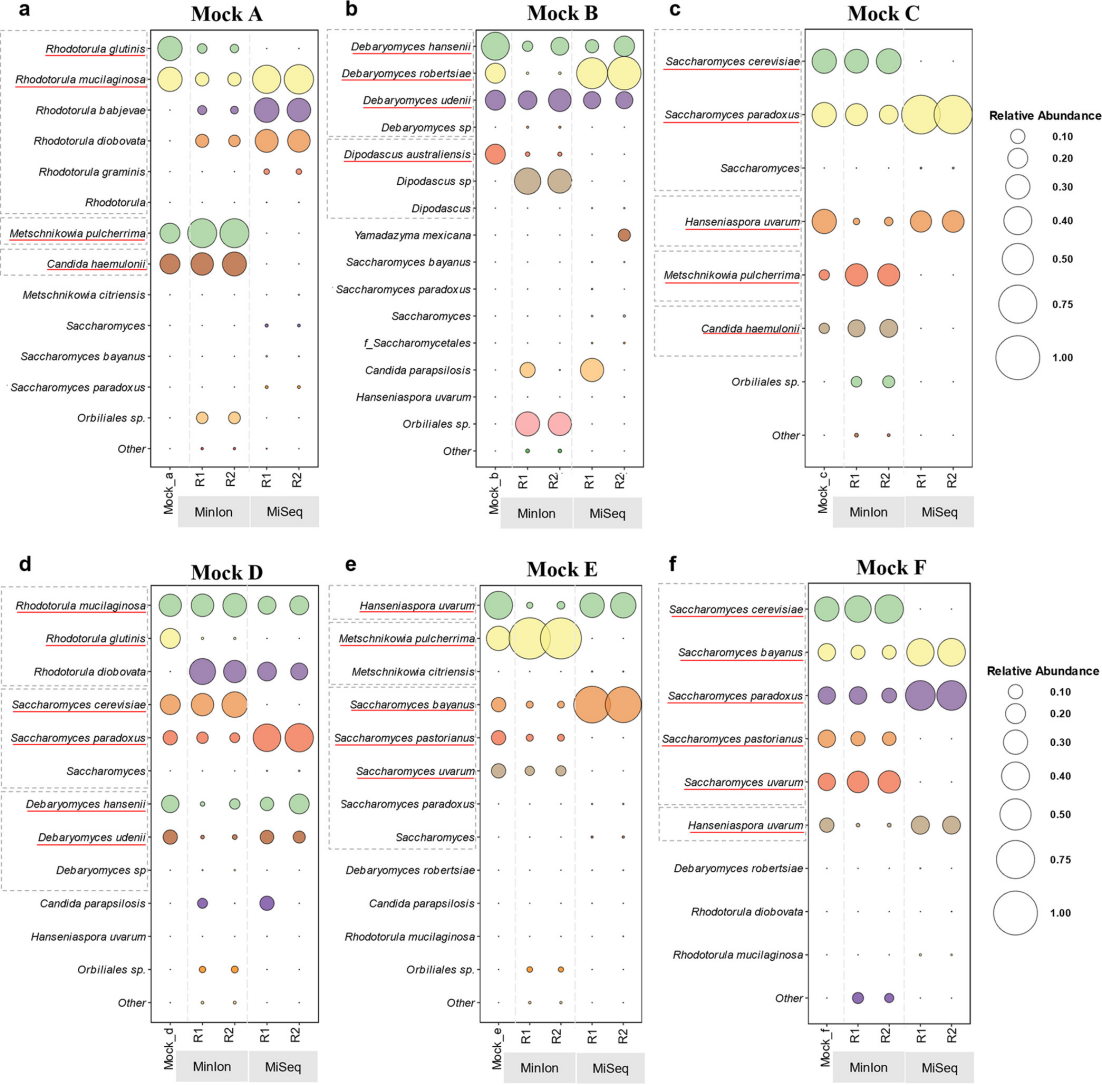
Comparison of mock communities’ fungal diversity at the species level, obtained by MinION and Illumina MiSeq, considering a dedicated reference database. Each panel shows the abundance of species found within a mock community, expressed with circles of different magnitudes that increase proportionally with the increase of relative abundance from 0 to 1. The database used for the mapping is composed only of ITS sequences belonging to the species that were identified with the first mapping against the full ITS database. For each mock community, the results are summarized in five columns. The left-most column of each panel represents the supposed abundance present in the mock community, and the following two columns represent the two biological replicates (labeled R1 and R2) obtained by MinION, while the last two are those obtained by MiSeq.

**(i) Performance of dedicated reference databases at the species level.** The MinION dedicated database was composed of ITS and LSU sequences of the species identified by the three full databases. Similarly, the Illumina dedicated database was generated only with the ITS2 sequences, obtained from UNITE, of the species identified in the first round of mapping against the full databases. The results showed that the use of dedicated databases mitigates the insurgence of ghost species, leading to better outcomes both taxonomically (i.e., the matching of species present and observed) and quantitively. MinION identified all of the organisms present in the mixture at the species level, while Illumina never detected *R. glutinis*, *D. australiensis*, *M. pulcherrima*, *C. haemulonii*, and the differences among the species of the *Saccharomyces sensu stricto* group (Fig. 5).

**FIG 6:**
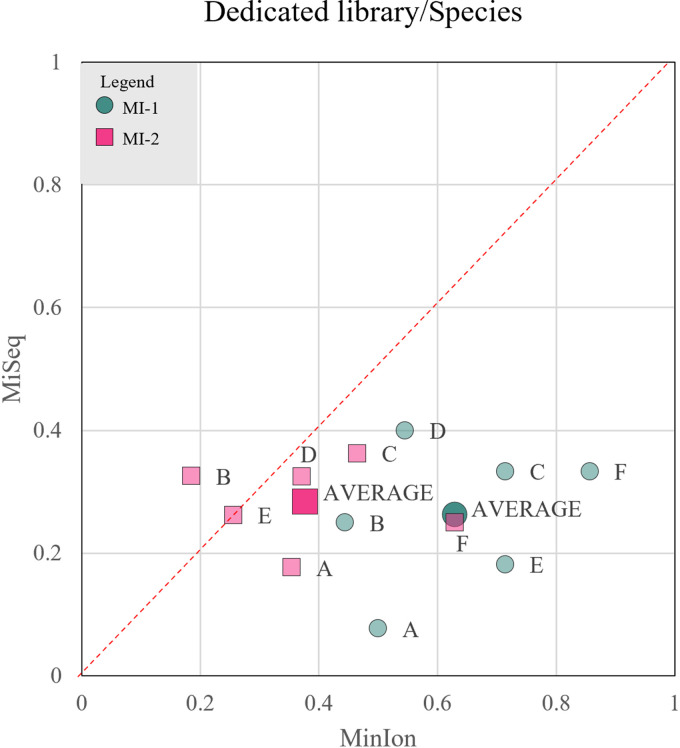
Comparison of the performance of MinION and MiSeq at the species level with a qualitative and a quantitative approach, considering a dedicated database. In order to compare the performance of the two sequencing platforms, two scores were developed to evaluate the matching of estimated and observed values from both a qualitative (MI-1 [pink squares]) and quantitative (MI-2 [dark blue-green circles]) viewpoint. Each mock community (labeled A, B, C, D, E, and F) is characterized by the two scores calculated at the species level for both MinION (*x* axis) and MiSeq (*y* axis), after the second step of mapping against a dedicated reference database. The average value for each score, obtained considering all mock communities, is characterized by a bigger marker.

On the contrary, long sequences mapped against restricted databases allowed the clear separation of closely related species like those of the genus *Saccharomyces* (Fig. 5c to f). MinION combined with the dedicated database tended to overestimate the abundance of *M. pulcherrima* (Fig. 5a, c, and e) but did not introduce the ghost *Metschnikowia* species, as with the full reference database (Fig. 3). Furthermore, dedicated libraries did not ameliorate the estimation of *H. uvarum* and the species of the genus *Debaryomyces*, which remained below the expected values. Similarly, *Rhodotorula* species were underestimated by MinION (Fig. 5a) due to two misclassifications that lowered the abundances of the two expected species. On the contrary, Illumina overestimated *Rhodotorula* species, while introducing ghosts of the same genus. S. paradoxus and S. bayanus were strongly overestimated with Illumina, because they were the only species of *Saccharomyces* to be identified. To summarize, MinION strongly outscored Illumina with both matching indices; in fact, the former obtained an MI-1 score of 0.63 and MI-2 score of 0.28, while the latter had scores of 0.26 and 0.28, respectively. These figures indicate that the introduction of dedicated reference database improved mostly the qualitative aspects of the identifications, whereas the quantification still needs further improvements (Fig. 6).

**(ii) Similar performance of ITS- versus LSU-based libraries with dedicated libraries with MinION.** The analysis of the abundances obtained from different markers was carried out considering only MinION sequences mapped against the dedicated database with both ITS and LSU sequences. The two barcodes returned similar proportions of the prevalent yeasts present in the mock communities, while differing in the taxonomic placement of some of them (Fig. S6).

Both ITS and LSU strongly overestimated *M. pulcherrima* and underestimated *D. hansenii* and *H. uvarum*. Species of *Saccharomyces sensu stricto* were clearly distinguished by the two markers, with a little overestimation of S. cerevisiae (Fig. S6d and f). Although the use of dedicated databases mitigated the identification of ghost species, ITS sequences were more prone than LSU to misclassification of some organisms. In fact, a considerable percentage of the ghost *Orbiliales* species was detected with ITS in almost every mock community. On the contrary, the conservative nature of LSU complicated the differentiation among closely related species like *Debaryomyces*, which are phylogenetically close (27) (Fig. S6b and d).

It is important to highlight that some level of biased estimation can be probably due to PCR amplification. Mock communities A, C, and E, showed a massive increase of *M. pulcherrima* abundance, which was linked to large underestimation of other species combined with it in the mixture (i.e., species of *Rhodotorula* [Fig. S6a] or *Saccharomyces* [Fig. S6e]), which in other mock communities were well estimated. This result could be explained considering that the *M. pulcherrima* ITS, with its ca. 250 bp, is one of the shortest ITSs among yeasts and could have outperformed longer sequences of the mock community in the amplification step as suggested from the known phenomenon that amplification of shorter DNA fragments is favored during PCR (28).

## DISCUSSION

Mock mixtures are a simulation-based approach to check the quality of the species abundance generated by amplicon-based metagenomics. The mixture can be generated by mixing the cells, the genomic DNA, or the amplicons in the correct percentages. We decided to mix purified genomic DNA in order to exclude all issues derived from differential DNA extraction of the various species, but to include the PCR amplification step in order to maintain a situation mimicking real-world procedures. Moreover, it was decided to amplify together the ITS and the LSU regions to avoid unbalanced amplification of the two marker regions. However, this choice could not prevent DNA of different species from being amplified at different rates due to scarce similarity of the primer to the target (15) or to the different lengths of the amplicons producing a competition favoring the shorter sequences (29). It must be considered, however, that this is an unavoidable problem inherent in metabarcoding, especially when using markers of different lengths (29–31). Another problem linked to the current situation in fungal taxonomy, and therefore in metabarcoding, is the multigene nature of the rRNA markers and the intragenomic variability of the repeats (32–35). All of these factors can unbalance the relative amounts of amplicons, leading to a number of sequences not proportional to the cell densities (36, 37), are somehow intrinsic to the biochemical procedures and to the specific nature of multigene markers of rDNA, and are hard to change as long as ITS and LSU are the most important markers in fungal taxonomy (6, 38). The settings used aimed at reducing these problems, without pretending to eliminate them, and the results showed that a careful manipulation can produce very high reproducibility among the replicates (Fig. 1). In order to make the simulations as close as possible to real environmental conditions, species compositions were varied to have the presence of prevalent species and low concentrations of species.

The specific aim of this work was to analyze the effects of the type and size of the database used in a bioinformatic pipeline on attributing reads to various species. Given the scarce taxonomic resolution of the available barcoding markers, we postulated that the presence of many related species is likely to decrease the accuracy of the identifications and therefore the abundance estimations. This concept was successfully tested in a previous paper by using a full reference database for a primary identification to produce the candidate taxa that then populated a dedicated reference database for the final, more accurate identification (24). The results of this paper showed that the dedicated databases were able to correctly identify single strains and to give an estimate of the similarity very close to the expected abundances of the mock communities and that the use of dedicated databases was able to produce abundances relatively close to those expected in the various mock communities.

The importance of highly curated and somehow focused reference databases has already been investigated at the level of all fungi and of pathogenic fungi in particular (39), showing that many sequences in public databases are too short or inaccurate or are derived from strains far away from the center of distribution of the species or from its type strain to be really good representatives (40). In addition to these problems, currently used markers in fungal taxonomy and barcoding cannot guarantee a full taxonomic resolution (25, 41) as single gene protein-encoding markers (42, 43), for which, however, universal anchoring positions are difficult to find, producing different levels of amplification in diverse taxa (44). Within these two rDNA markers, in this paper we showed that, in general, ITS-based reference databases work better than LSU ones. However, some taxonomic complexes with closely related species, such as *Saccharomyces sensu stricto* and the species of the genus *Debaryomyces*, yielded problems due to the lack of resolution of the ITS. Furthermore, there are classification problems due to the presence of hybrids or to unresolved taxa leading to spurious identifications, such as S. cerevisiae*/*S. paradoxus present in the CBS database. In general, ITS was more accurate than LSU, but ITS was also more prone to produce ghost species. On the other hand, LSU had the opposite behavior, leading to less accuracy but also to lower production of ghost species, implying that the former has the sensitivity for which it has been elected as a universal marker (6) but not necessarily the accuracy for species-specific identification when species are phylogenetically close. The simultaneous use of both markers showed only slight general improvements but was advantageous when close species (as those of *Saccharomyces* and *Debaryomyces*) have to be dissected. These observations suggest that, as long as better markers will be available, the use of ITS alone is justified (45), although it is important to be aware of its limitations, especially when the sole ITS2 portion is used, as normally happens with the Illumina platform (46). The fact that the databases available contain often partial and too short sequences is a further aspect hampering correct identification and calls for the building of reference databases with full sequences, possibly including the whole region spanning the ITS and LSU to increase taxonomic resolution at the species level, which is probably the preferential choice in many studies regarding the mycobiome. A coordinated effort by researchers working in the field could convoy high-quality ITS-LSU sequences in public focused databases to hasten the attainment of this goal.

### Conclusions.

Metagenomic analysis mostly employs short fragments of rDNA sequences for the identification of microbial communities. The choice of the region to amplify together with the use of different databases could cause discrepancies among results. Long-read technologies can mitigate biases due to length, primer choices, and copy number variation, which constitute relevant limitations in this type of analysis. Moreover, the two-step procedure proposed in this paper avoided “ghost” species in most cases and could guarantee that their abundance is normally low. This opens the question of the minimum level of abundance for a taxon to be considered really present in a community, but at the current state of the art, this is probably beyond the possibilities of the markers available. More and better-performing markers will be a key aspect for future metagenomics, but their use will not be possible as long as convenient procedures and large data sets will be prepared. Even considering using a “shotgun” NGS procedure, these markers will be indispensable, making their development more urgent.

In general, it is clear that for the efficient use of next-generation sequencing in metabarcoding, next-generation reference databases have to be generated by a community effort.

## MATERIALS AND METHODS

### Species and growth conditions.

The strains used in the study were initially cultivated in plate with YPDA medium (1% yeast extract, 1% peptone, 2% dextrose, 1.8% agar). A colony for each sample was inoculated in YPD medium and grown in shaking mode at 25°C for 24 h. The strains used are listed in Table 1. Strains were cultivated in duplicate to have statistically significant biological replicates.

**TABLE 1:** List of strains used for the mock communities^*a*^

Species	Strain
Debaryomyces hansenii	CBS 5637
	CBS 767
Debaryomyces robertsiae	CBS 4288
Debaryomyces udenii	CBS 7056
Dipodascus australiensis	LCF 1641
Hanseniaspora uvarum	CBS 314
	LCF 1073
Candida haemulonii	CBS 5149
Metschnikowia pulcherrima	CBS 5833
Rhodotorula glutinis	CBS 20
Rhodotorula mucilaginosa	CBS 316
	CBS 326
Saccharomyces bayanus	CBS 380
Saccharomyces cerevisiae	CBS 1171
	LCF 520
Saccharomyces paradoxus	CBS 432
Saccharomyces pastorianus	CBS 1538
Saccharomyces uvarum	CBS 395

a All of the strains used in the mock communities are listed by species name and strain collection ID. All LCF strains are part of the laboratory internal strain collection.

### DNA extraction, mock community preparation, and PCR amplification.

Liquid cultures were collected and transferred into extraction tubes, which were centrifuged at 4,500 rpm for 3 min to pellet the cells. The supernatant was removed, and cells were washed with 5 mL of nuclease-free water (Sigma-Aldrich). The procedure was repeated twice. A 0.5-mL concentration of nuclease-free water was added to the dried pellet, together with glass beads, and cells were resuspended by vortexing. The same volume of lysis buffer (2% Triton X-100, 1% SDS, 100 mM NaCl, 1 mM EDTA) was pipetted into the suspension. Mechanical lysis was carried out by shaking the suspension on FastPrep homogenizers (MP Biomedicals) at 6.0 m/s for 30 s. Lysates were centrifuged at 4,500 rpm for 3 min. Subsequently, 0.7 mL of supernatant was collected and transferred into clean microcentrifuge tubes. DNA purification was completed according to the procedure suggested by FastDNA spin kit for Soil (MP Biomedicals) from point 6 on. The DNA extracted was quantified by measuring absorbance at 260 nm with a NanoDrop spectrophotometer (Thermo Scientific). For each sample, three measures were picked and the average value was taken into consideration for the further step. Six different mock communities were built by mixing precise amounts of DNA as reported in Table 2.

**TABLE 2:** Compositions of the six mock communities^*a*^

Mock	Species	Strain	Expected abundance (%)
A	Candida haemulonii	CBS 5149	20
	Metschnikowia pulcherrima	CBS 5833	20
	Rhodotorula glutinis	CBS 20	15
		CBS 2366	15
	Rhodotorula mucilaginosa	CBS 316	15
		CBS 326	15

B	Debaryomyces hansenii	CBS 5637	20
		CBS 767	20
	Debaryomyces robertsiae	CBS 4288	20
	Debaryomyces udenii	CBS 7056	20
	Dipodascus australiensis	LCF 1640	20

C	Saccharomyces cerevisiae	LCF 520	30
	Saccharomyces paradoxus	CBS 432	30
	Hanseniaspora uvarum	CBS 314	15
		LCF 1073	15
	Metschnikowia pulcherrima	CBS 5833	10

D	Rhodotorula mucilaginosa	CBS 316	25
	Rhodotorula glutinis	CBS 20	20
	Saccharomyces cerevisiae	CBS 1171	20
	Saccharomyces paradoxus	CBS 432	10
	Debaryomyces hansenii	CBS 767	15
	Debaryomyces udenii	CBS 7056	10

E	Hanseniaspora uvarum	CBS 314	40
	Metschnikowia pulcherrima	CBS 5833	30
	Saccharomyces bayanus	CBS 380	10
	Saccharomyces pastorianus	CBS 1538	10
	Saccharomyces uvarum	CBS 395	10

F	Saccharomyces bayanus	CBS 380	15
	Saccharomyces cerevisiae	CBS 1171	15
		LCF 520	15
	Saccharomyces paradoxus	CBS 432	15
	Saccharomyces pastorianus	CBS 1538	15
	Saccharomyces uvarum	CBS 395	15
	Hanseniaspora uvarum	CBS 314	10

a For each mock community, the table reports the strains used with their abundance in the final mixture.

This step was performed twice to have duplicated mock communities to ensure two biological replicates. Mock communities were prepared considering both quantitative and qualitative two levels of evaluation. From a quantitative point of view, different abundance values were considered to assess whether they would be maintained through the process. Furthermore, species and strains were assembled considering different phylogenetic distances (see Table S1 in the supplemental material) to test the resolution power of MinION sequencing technology in discriminating closer species. Yeast species were chosen for simulation of natural interactions in real environments (e.g., fermentation, food, or soil).

The marker genes, including ITS1, 5.8S, ITS2 rDNA genes, and the D1/D2 domain of the LSU of each of the mock communities, were amplified in triplicate. The master mix used was TaKaRa *Taq* DNA polymerase (TaKaRa Bio, Inc.), with the primers ITS1 (5′-TCCGTAGGTGAACCTGCGG) and NL4 (5′-GGTCCGTGTTTCAAGACGG) (47). Here, the amplicons obtained after this first PCR will be called round 1 products.

The amplification protocol was carried out as follows: initial denaturation at 94°C for 3 min, followed by 30 amplification cycles of 94°C for 1 min, 54°C for 1 min, and 72°C for 1 min, and then a final extension at 72°C for 5 min. Finally, we had 48 ITS-D1/D2 amplicons (3 technical replicates for each mock community for the two biological replicates), which were checked on 1% agarose gel.

### Library preparation and MinION sequencing.

Round 1 products were subjected to a tagging step that consisted of 3 min of denaturation at 95°C followed by 25 cycles of 95°C for 30 s, 68°C for 30 s, and 72°C for 1 min. The primers used were ITS1 and NL4 tailed with the following universal sequences: ITS1, 5′-**TTTCTGTTGGTGCTGATATTGC**[TCCGTAGGTGAACCTGCGG]-3′; NL4, 5′-**ACTTGCCTGTCGCTCTATCTTC**[GGTCCGTGTTTCAAGACGG]-3′. Universal primers (sequence in lightface between brackets) were fused with the specific tags reported in boldface.

PCR products were size selected by being cleaned up with 0.7× volume of Ampure XP (Beckman Coulter, Brea, CA, USA). A 200-fmol concentration of each sample was used for barcoding step, according to the ligation sequencing kit 1D (SQK-LSK109) and the PCR barcoding expansion pack 1-96 (EXP-PBC096) protocol (Oxford Nanopore Technologies, Oxford, United Kingdom). After a purification step with 0.7× Ampure XP, a pooled barcoded library was prepared by mixing 10.46 ng of DNA per sample to reach a final concentration of 1 μg of DNA in 47 μL of nuclease-free water. The library was end repaired and adapted for Nanopore sequencing by using the NEBNext Ultra DNA library preparation kit. A 50-fmol concentration of product was loaded onto an R9.4.1 flow cell. The quantification steps were carried out with a NanoDrop 1000 (Thermo Scientific). Reads were base called on-instrument using the Guppy v.4.2.2 GPU base caller (Oxford Nanopore Technologies). MinION sequences are stored in the SRA (Table S2).

### Library preparation for Illumina MiSeq sequencing.

Round 1 products were also amplified using primers specific for the Illumina platform. Universal primers ITS3f and ITS4r were tailored with the following tags: ITS3f, 5′-**TCGTCGGCAGCGTCAGATGTGTATAAGAGACAG**[GCATCGATGAAGAACGCAGC]-3′; ITS4r, 5′-**GTCTCGTGGGCTCGGAGATGTGTATAAGAGACAG**[TCCTCCGCTTATTGATATGC]-3′. Universal primers (sequence in lightface between brackets) were fused with the specific tags reported in boldface.

The amplification protocol was carried out as follows: initial denaturation at 94°C for 1 min, followed by 25 amplification cycles of 94°C for 30 s, 55°C for 30 s, and 68°C for 45 s, and a final extension at 68°C for 7 min.

The amplificons tagged for MiSeq sequencing were sent to BMRGenomics (Padua, Italy) for further processing. MiSeq sequences are stored in the SRA archive (Table S3).

### Sequence analysis pipeline and reference databases.

**(i) MinION.** The sequence analysis pipeline worked in a *conda* environment built in Ubuntu. Filtering processes of raw reads was carried out by using the function *seqtk*, which removed sequences below 400 bp and greater than 1,500 bp. Filtered reads were merged in one file that was used as input for the alignment program *minimap2* (48). Such a tool allows the alignment of sequences against a large reference database.

The algorithm was tuned to support the alignment of long noisy reads by using the option *map-ont*, which uses ordinary minimizers as seeds. Two different classes of databases were used: full and dedicated databases. The former databases are those commonly used in metabarcoding studies and include a comprehensive panel of sequences for the identification of ideally the entire spectrum of Fungi. The full databases used in this study are the General Release reference database from UNITE (49) and the CBS reference database from Westerdijk Fungal Biodiversity Institute. General Release was downloaded from the UNITE database, and it comprises 58,440 ITS sequences among the RepS/RefS of all species hypotheses (SHs). The second full database was built with 34,683 ITS and LSU D1/D2 separated sequences taken from CBS collection. The other class of databases is the dedicated database, which is a restricted form that comprises ITS and LSU sequences of all of the species that were identified from all of the mock communities with a first round of mapping against the full database. SAM files that resulted from the alignment step were further processed with programs of the SAMtools package (50) up to a tab-delimited table.

The relative abundances that resulted from the mapping are provided as supplemental data.

**(ii) MiSeq.** The bioinformatic processing of raw sequences was done following the procedure developed by Callahan et al. (51) to obtain amplicon sequence variants (ASVs) from the raw reads (R package version 1.16.0, with the trunLen parameter set to 260 bp for forward reads and 190 bp for reverse reads). ASVs that originated from ITS2 sequences were first classified using only the full database UNITE. After the first round of classification, the sequences of the species identified were selected and used for the construction of the dedicated database, which was used for a second round of classification of the raw reads. The relative abundances that resulted from the mapping are provided as supplemental data.

### Data analysis.

**(i) General.** Macros written in MS Excel were used to prepare tables, which were subjected to analyses in R (52).

A first step consisted in finding the number of unique reads mapped to a reference. The function *samtools flagstats* counts the number of alignments for each FLAG type giving the values of primary, secondary, and supplementary reads mapped on a specific reference. By subtracting those values in the reported order, we obtained the number of reads uniquely mapped to a reference in the database. Relative abundances were calculated as the ratio of unique reads mapped to a reference on the total reads mapped to the database (calculated as the sum of unique reads of all the references in the database). Microbiome data were stored, analyzed, and graphically displayed with the R package *microeco*. Similarly, correlation coefficients were computed in the R environment, using the function *cor()*. Coefficients were calculated by both the Spearman and Pearson methods.

**(ii) Matching indices.** To compare the accuracies of the two sequencing methods, two different indices were calculated: one qualitative (matching index 1 [MI-1]) and one quantitative (MI-2). The calculation of the two indices was achieved with the same formula (equation 1), although the factors are qualitative for MI-1 and quantitative for MI-2 as detailed below:

(1) $$\text{MI}=\frac{\sum_{i=0}^{n}{\text{TP}}_{i}}{\sum_{i=0}^{n}[{\text{TP}}_{i}+{\text{FP}}_{i}+{\text{FN}}_{i}]}$$

MI-1 was calculated by dividing the total number of true-positive (TP) identifications by the number of false-positive (FP), false-negative (FN), and true-positive (TP) identifications (equation 1) for each mock community, where each term was calculated as follows. (i) TP*_i_* indicates that when a species present in the mock community is correctly identified (regardless of its relative abundance), it is considered a true positive and is given the value 1. (ii) FP*_i_* indicates that when a species absent in the mock community is present in the final identification (regardless of its relative abundance), it is considered a false positive and is given the value 1. (iii) FN*_i_* indicates that when a species present in the mock community is not found to be present in the final identification, it is considered false negative and is given the value 1.

Matching index 2 (MI-2) was obtained by the same formula, but its factors are defined as follows. (i) TP*_i_* is the lower value between the observed and expected relative abundances of the *i*th species. (ii) FP*_i_* is the difference between the observed and the expected relative abundances of the *i*th species if the value is greater than 0, calculated as |observed − expected|. (iii) FN*_i_* is the difference between the observed and the expected relative abundances of the *i*th species if the value is less than 0, calculated as |observed − expected|.

True-negative (TN) results could not be considered in the context of these experiments because they would correspond to the true absence of all known species not included in the mock communities, leading to a seriously biased index.

### Data availability.

The data that support the findings of this study are openly available in the SRA archive under BioProject accession no. PRJNA862129 (MinION data) and PRJNA862334 (MiSeq data).
