# Supplementary material for: MinION Sequencing of Yeast Mock Communities To Assess the Effect of Databases and ITS-LSU Markers on the Reliability of Metabarcoding Analysis
Source: Microbiol Spectr. 2022 Dec 15;11(1):e01052-22. doi: 10.1128/spectrum.01052-22 (PMC9927109; doi:10.1128/spectrum.01052-22)
Supplement: Supplemental file 1 — Supplemental material. Download spectrum.01052-22-s0001.pdf, PDF file, 0.5 MB [file spectrum.01052-22-s0001.pdf]

Supplementary Figure 1. Comparison of fungal diversity of mock communities at genus level, obtained with MinION and Illumina MiSeq, considering full libraries (ITS).

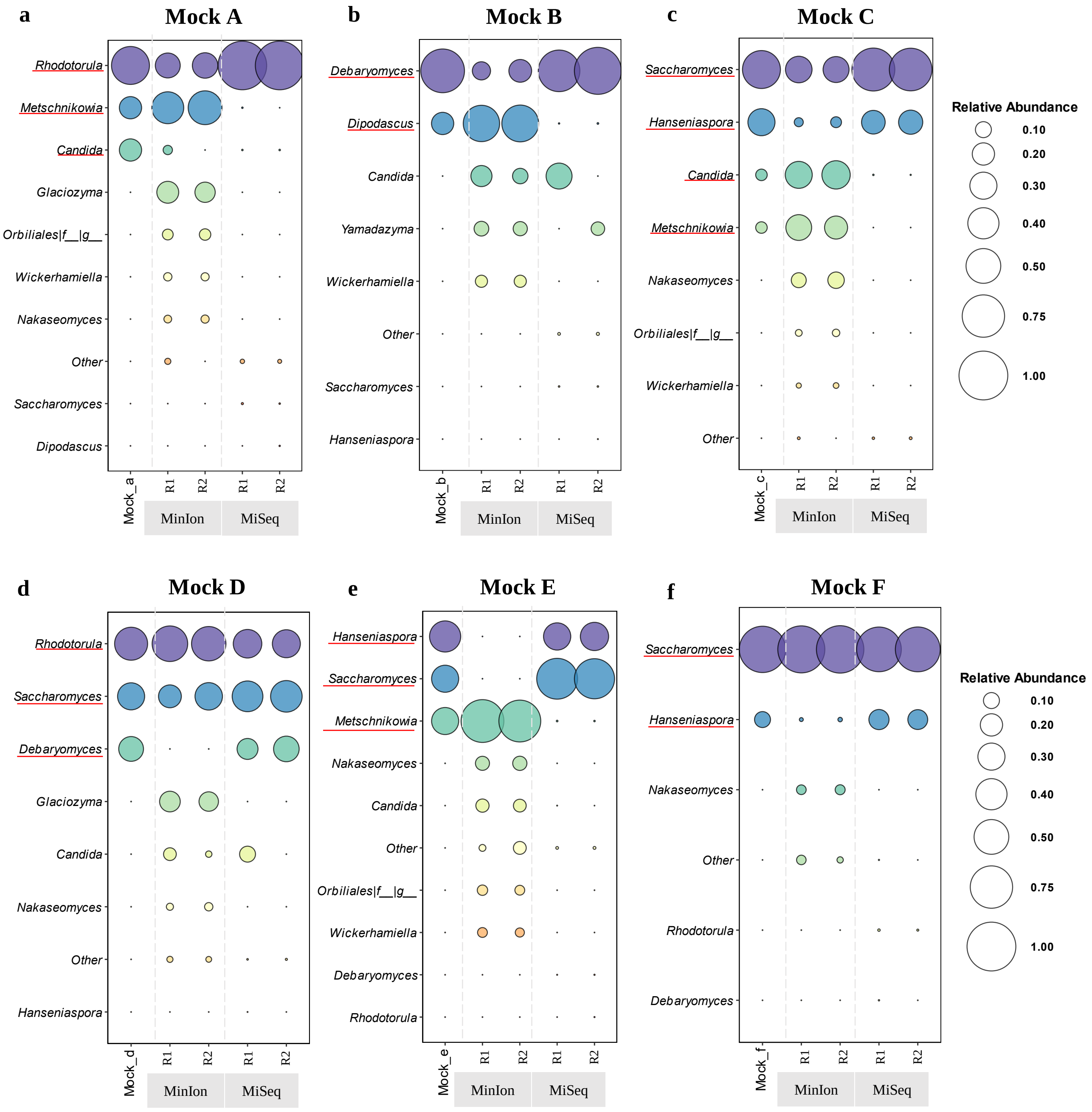

Each panel shows the abundance of genera found within a mock community, expressed with circles of different magnitude that increase proportionally with the increase of relative abundance from 0 to 1.

The library used for the mapping is composed only of ITS sequences, in other to compare the two different sequencing methods.

For each mock, the results are summarized in five columns. The left-most column of each panel represents the supposed abundance present in the mock, the following two columns represent the two biological replicates (labelled with R1 and R2) obtained with MinION while the last two are those obtained with MiSeq.

Supplementary Figure 2. Comparison of the performance of MinION and MiSeq, at genus level, with a qualitative and a quantitative approach, considering a full ITS library.

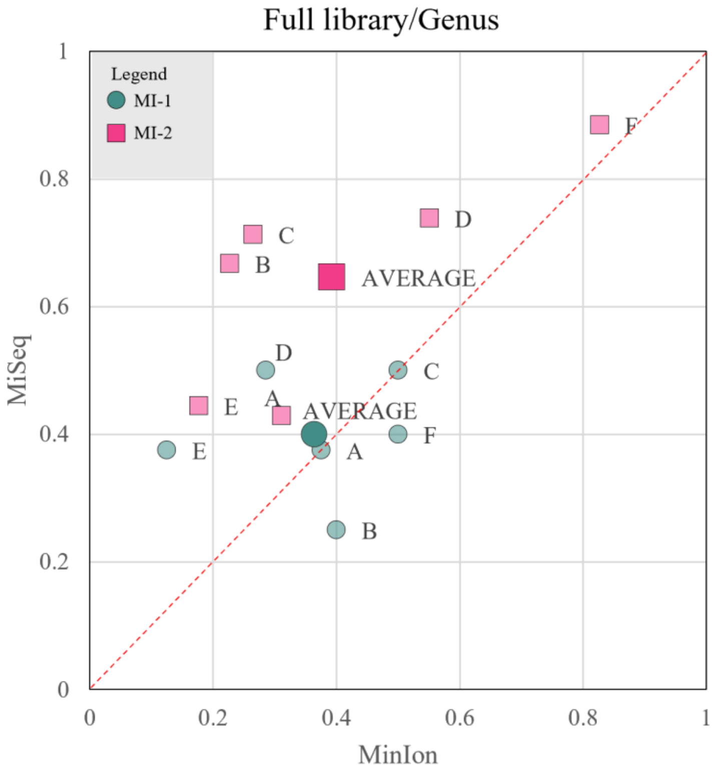

In order to compare the performance of the two sequencing platforms, two scores were developed to evaluate the matching of estimated and observed values from both a qualitative (MI-1, in pink squares) and quantitative (MI-2, in dark blue-green circles) viewpoint.

Each mock (labelled with the letters A, B, C, D, E and F) is characterized by the two scores calculated, at the genus level, both for MinION (x-axis) and MiSeq (y-axis), considering a full ITS library. The average value for each score, obtained considering all mocks, is characterized by a bigger marker.

Supplementary Figure 3. UNITE library reference sequences length distribution.

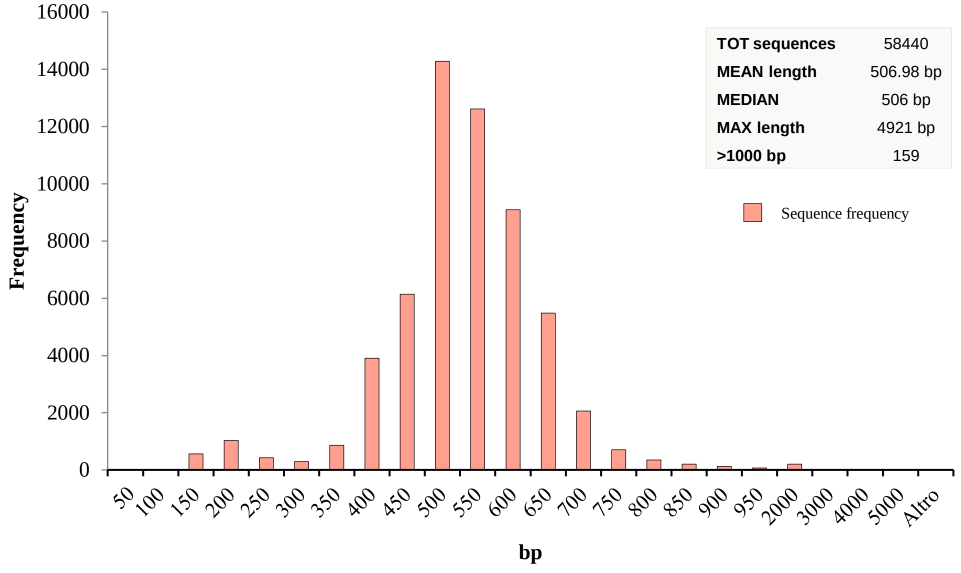

The distribution of reference sequences length spans from 150 to 5000 bp. In the box, the total number of sequences, mean, median and maximum length are also presented.

Supplementary Figure 4. Phylogenetic tree of UNITE reference sequences

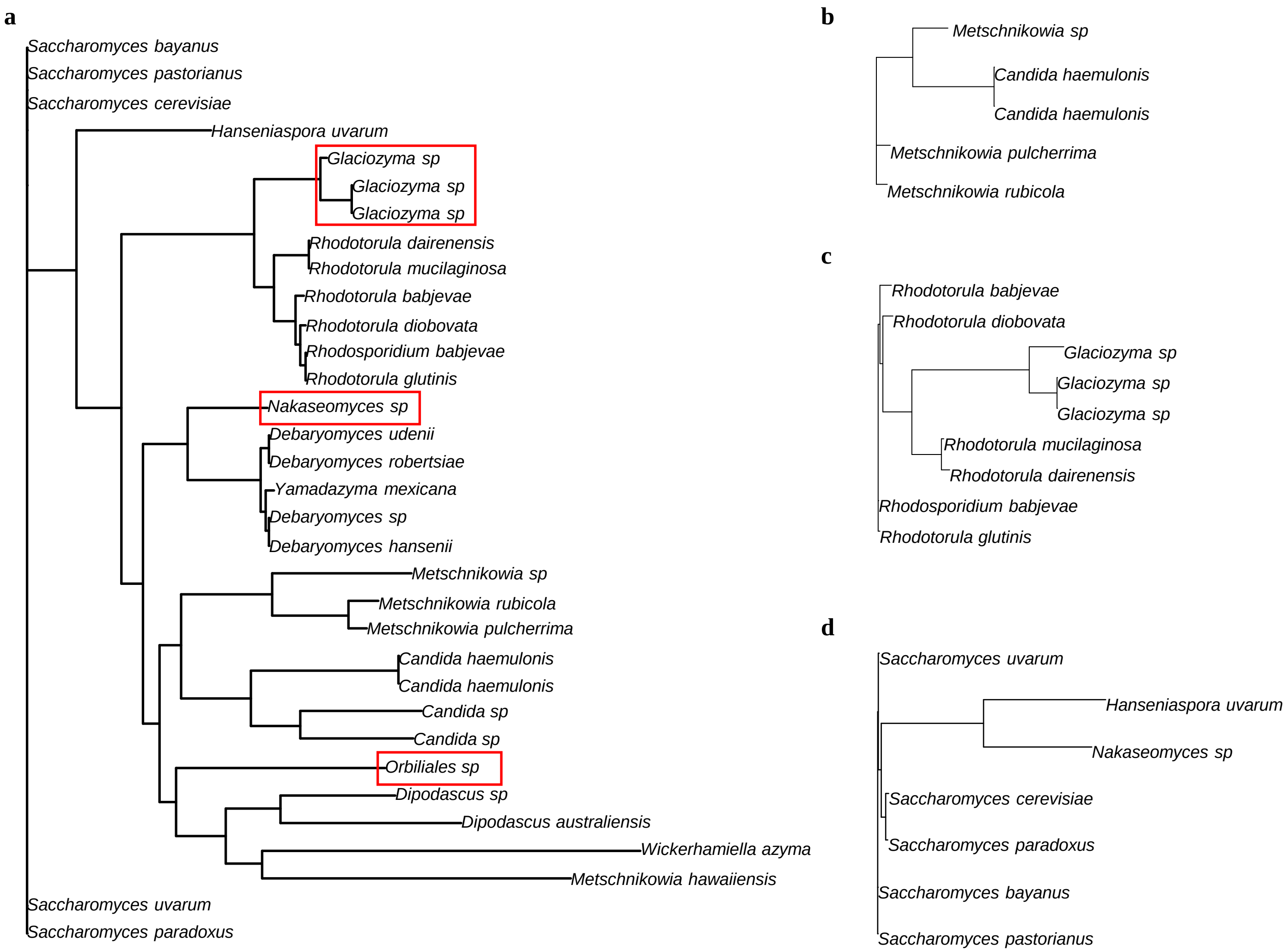

Phylogenetic relationships among organisms identified with full databases. Panel a shows a comprehensive tree of all detected species while panels b, c and d are restricted to one genus.

Supplementary Figure 5. Comparison of the performance of MinION and MiSeq, at species level, with a qualitative and a quantitative approach, considering a dedicated library.

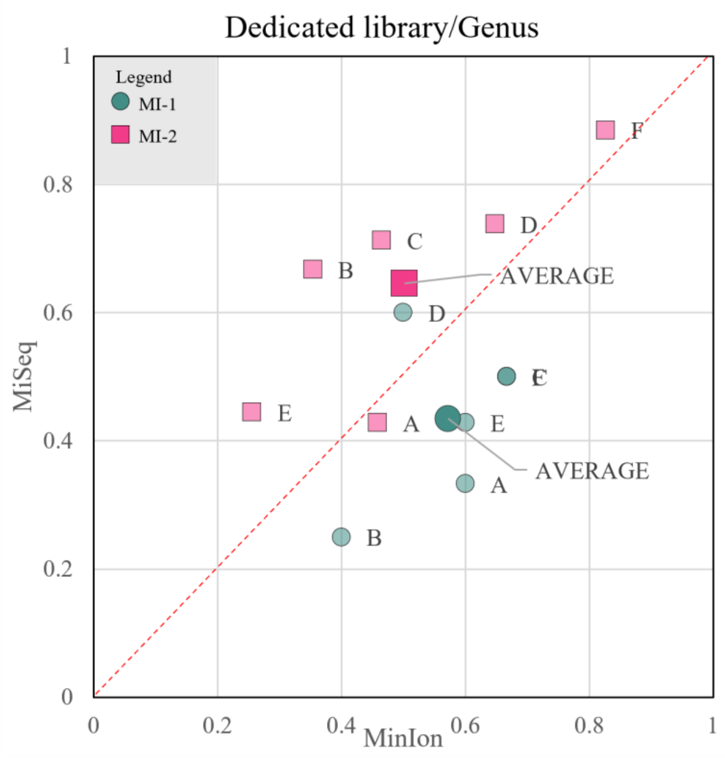

In order to compare the performance of the two sequencing platforms, two scores were developed to evaluate the matching of estimated and observed values from both a qualitative (MI-1, in pink squares) and quantitative (MI-2, in dark blue-green circles) viewpoint.

Each mock (labelled with the letters A, B, C, D, E and F) is characterized by the two scores calculated, at the genus level, both for MinION (x-axis) and MiSeq (y-axis), after the second step of mapping against a dedicated library. The average value for each score, obtained considering all mocks, is characterized by a bigger marker.

Supplementary Figure 6. Fungal diversity of mock communities considering dedicated libraries.

Each panel shows the abundance (y-axis) of species found within a mock community, obtained by mapping the sequences against a dedicated library after the first identification process against the full library. Both ITS and LSU barcodes from the CBS library were considered. For each barcode there are two columns that represent the two biological replicates (labelled with R1 and R2). The right-most column of each panel represents the mock expected abundance. Species present in the mock community are written in red, while the black ones are those identified by the mapping without being added initially.

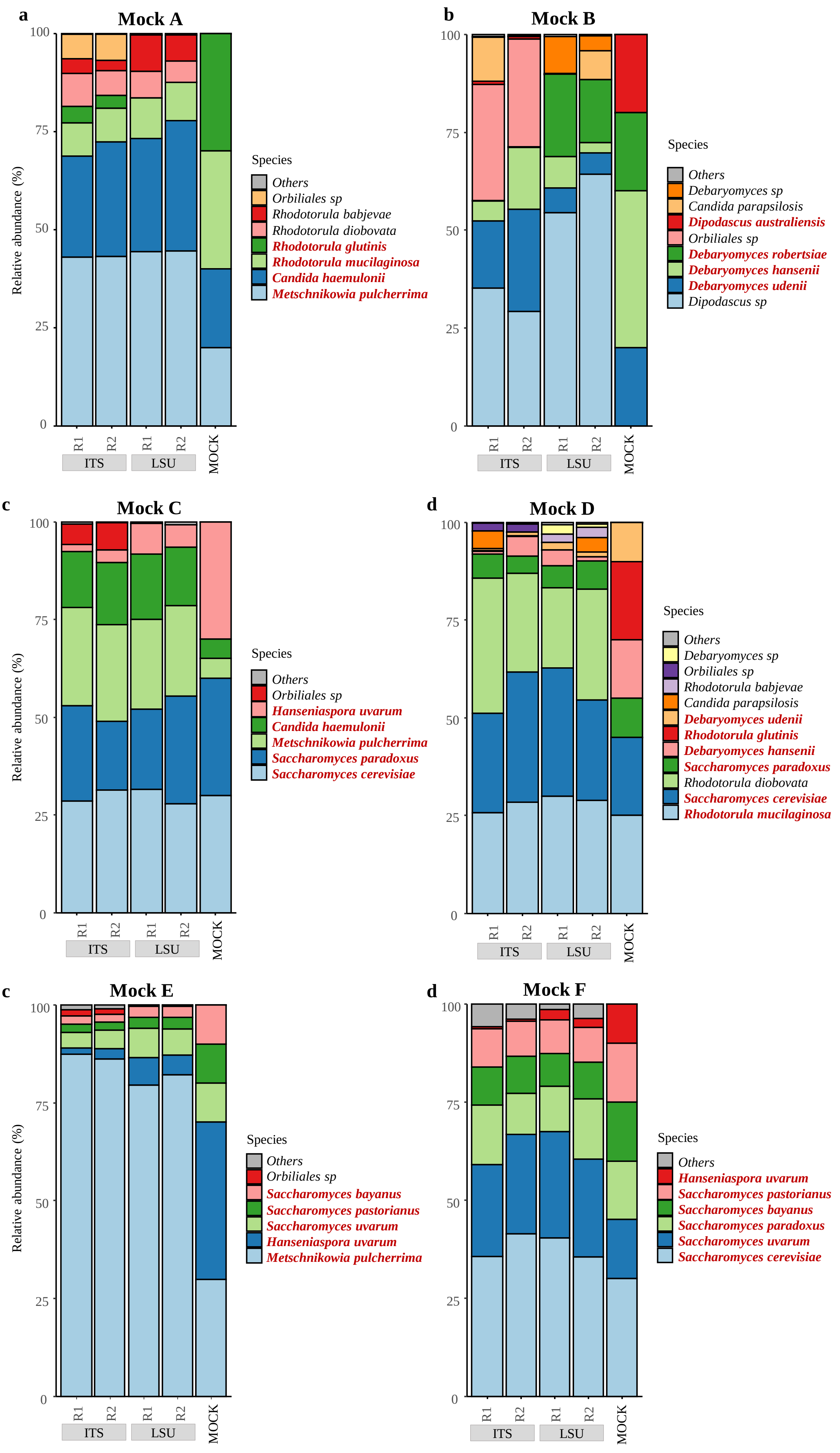
a
Mock A
Relative abundance (%)
100
75
50
25
0
R1
R2
R1
R2
ITS
LSU
MOCK
Species
Others
Orbiliales sp
Rhodotorula babjevae
Rhodotorula diobovata
Rhodotorula glutinis
Rhodotorula mucilaginosa
Candida haemulonii
Metschnikowia pulcherrima
b
Mock B
Species
Others
Debaryomyces sp
Candida parapsilosis
Dipodascus australiensis
Orbiliales sp
Debaryomyces robertsiae
Debaryomyces hansenii
Debaryomyces udenii
Dipodascus sp
c
Mock C
Species
Others
Orbiliales sp
Hanseniaspora uvarum
Candida haemulonii
Metschnikowia pulcherrima
Saccharomyces paradoxus
Saccharomyces cerevisiae
d
Mock D
Species
Others
Debaryomyces sp
Orbiliales sp
Rhodotorula babjevae
Candida parapsilosis
Debaryomyces udenii
Rhodotorula glutinis
Debaryomyces hansenii
Saccharomyces paradoxus
Rhodotorula diobovata
Saccharomyces cerevisiae
Rhodotorula mucilaginosa
c
Mock E
Species
Others
Orbiliales sp
Saccharomyces bayanus
Saccharomyces pastorianus
Saccharomyces uvarum
Hanseniaspora uvarum
Metschnikowia pulcherrima
d
Mock F
Species
Others
Hanseniaspora uvarum
Saccharomyces pastorianus
Saccharomyces bayanus
Saccharomyces paradoxus
Saccharomyces uvarum
Saccharomyces cerevisiae

Supplementary table 1: distance matrix of the species included in the mock communities

Phylogenetic relationships among the species used in the experiment

	Candida haemulonii	*Debaryomyces hansenii*	*Debaryomyces robertsiae*	*Debaryomyces sp*	*Debaryomyces udenii*	*Dipodascus australiensis*	*Dipodascus sp*	*Hanseniaspora uvarum*	*Metschnikowia hawaiiensis*	*Metschnikowia pulcherrima*	*Rhodotorula glutinis*	*Rhodotorula mucilaginosa*	*Saccharomyces bayanus*	*Saccharomyces cerevisiae*	*Saccharomyces paradoxus*	*Saccharomyces pastorianus*	*Saccharomyces uvarum*
Candida haemulonii	0.0%	17.2%	16.7%	16.7%	16.7%	24.2%	28.3%	22.7%	28.8%	15.7%	29.3%	28.8%	21.2%	21.2%	21.2%	21.2%	21.2%
Debaryomyces hansenii	17.2%	0.0%	1.0%	0.5%	1.0%	27.8%	21.2%	15.2%	26.8%	20.2%	23.7%	23.7%	9.6%	9.6%	9.6%	9.6%	9.6%
Debaryomyces robertsiae	16.7%	1.0%	0.0%	0.5%	0.0%	28.3%	20.7%	15.2%	26.8%	19.7%	22.7%	23.7%	9.6%	9.6%	9.6%	9.6%	9.6%
Debaryomyces sp	16.7%	0.5%	0.5%	0.0%	0.5%	28.3%	21.2%	15.7%	27.3%	19.7%	23.2%	23.7%	10.1%	10.1%	10.1%	10.1%	10.1%
Debaryomyces udenii	16.7%	1.0%	0.0%	0.5%	0.0%	28.3%	20.7%	15.2%	26.8%	19.7%	22.7%	23.7%	9.6%	9.6%	9.6%	9.6%	9.6%
Dipodascus australiensis	24.2%	27.8%	28.3%	28.3%	28.3%	0.0%	24.2%	27.3%	33.3%	24.8%	33.8%	32.3%	27.3%	27.3%	27.3%	27.3%	27.3%
Dipodascus sp	28.3%	21.2%	20.7%	21.2%	20.7%	24.2%	0.0%	23.7%	26.8%	29.8%	28.3%	29.8%	23.7%	23.7%	23.7%	23.7%	23.7%
Hanseniaspora uvarum	22.7%	15.2%	15.2%	15.7%	15.2%	27.3%	23.7%	0.0%	26.8%	22.2%	24.8%	24.2%	12.1%	12.1%	12.1%	12.1%	12.1%
Metschnikowia hawaiiensis	28.8%	26.8%	26.8%	27.3%	26.8%	33.3%	26.8%	26.8%	0.0%	27.8%	34.9%	33.8%	26.8%	26.8%	26.8%	26.8%	26.8%
Metschnikowia pulcherrima	15.7%	20.2%	19.7%	19.7%	19.7%	24.8%	29.8%	22.2%	27.8%	0.0%	27.8%	28.3%	21.7%	21.7%	21.7%	21.7%	21.7%
Rhodotorula glutinis	29.3%	23.7%	22.7%	23.2%	22.7%	33.8%	28.3%	24.8%	34.9%	27.8%	0.0%	7.1%	21.7%	21.7%	21.7%	21.7%	21.7%
Rhodotorula mucilaginosa	28.8%	23.7%	23.7%	23.7%	23.7%	32.3%	29.8%	24.2%	33.8%	28.3%	7.1%	0.0%	21.7%	21.7%	21.7%	21.7%	21.7%
Saccharomyces bayanus	21.2%	9.6%	9.6%	10.1%	9.6%	27.3%	23.7%	12.1%	26.8%	21.7%	21.7%	21.7%	0.0%	0.0%	0.0%	0.0%	0.0%
Saccharomyces cerevisiae	21.2%	9.6%	9.6%	10.1%	9.6%	27.3%	23.7%	12.1%	26.8%	21.7%	21.7%	21.7%	0.0%	0.0%	0.0%	0.0%	0.0%
Saccharomyces paradoxus	21.2%	9.6%	9.6%	10.1%	9.6%	27.3%	23.7%	12.1%	26.8%	21.7%	21.7%	21.7%	0.0%	0.0%	0.0%	0.0%	0.0%
Saccharomyces pastorianus	21.2%	9.6%	9.6%	10.1%	9.6%	27.3%	23.7%	12.1%	26.8%	21.7%	21.7%	21.7%	0.0%	0.0%	0.0%	0.0%	0.0%
Saccharomyces uvarum	21.2%	9.6%	9.6%	10.1%	9.6%	27.3%	23.7%	12.1%	26.8%	21.7%	21.7%	21.7%	0.0%	0.0%	0.0%	0.0%	0.0%

Supplementary Table 2. MinION BioSample accessions

IDs of MinION sequences stored in SRA database for identifying each sample (BioProject ID PRJNA862129)

Accession	Sample Name	Organism
SAMN29939597	Mock_A_1_I	synthetic metagenome
SAMN29939598	Mock_A_1_II	synthetic metagenome
SAMN29939599	Mock_A_1_III	synthetic metagenome
SAMN29939600	Mock_A_2_I	synthetic metagenome
SAMN29939601	Mock_A_2_II	synthetic metagenome
SAMN29939602	Mock_A_2_III	synthetic metagenome
SAMN29939603	Mock_B_1_I	synthetic metagenome
SAMN29939604	Mock_B_1_II	synthetic metagenome
SAMN29939605	Mock_B_1_III	synthetic metagenome
SAMN29939606	Mock_B_2_I	synthetic metagenome
SAMN29939607	Mock_B_2_II	synthetic metagenome
SAMN29939608	Mock_B_2_III	synthetic metagenome
SAMN29939609	Mock_C_1_I	synthetic metagenome
SAMN29939610	Mock_C_1_II	synthetic metagenome
SAMN29939611	Mock_C_1_III	synthetic metagenome
SAMN29939612	Mock_C_2_I	synthetic metagenome
SAMN29939613	Mock_C_2_II	synthetic metagenome
SAMN29939614	Mock_C_2_III	synthetic metagenome
SAMN29939615	Mock_D_1_I	synthetic metagenome
SAMN29939616	Mock_D_1_II	synthetic metagenome
SAMN29939617	Mock_D_1_III	synthetic metagenome
SAMN29939618	Mock_D_2_I	synthetic metagenome
SAMN29939619	Mock_D_2_II	synthetic metagenome
SAMN29939620	Mock_D_2_III	synthetic metagenome
SAMN29939621	Mock_E_1_I	synthetic metagenome
SAMN29939622	Mock_E_1_II	synthetic metagenome
SAMN29939623	Mock_E_1_III	synthetic metagenome
SAMN29939624	Mock_E_2_I	synthetic metagenome
SAMN29939625	Mock_E_2_II	synthetic metagenome
SAMN29939626	Mock_E_2_III	synthetic metagenome
SAMN29939627	Mock_F_1_I	synthetic metagenome
SAMN29939628	Mock_F_1_II	synthetic metagenome

SAMN29939629	Mock_F_1_III	synthetic metagenome
SAMN29939630	Mock_F_2_I	synthetic metagenome
SAMN29939631	Mock_F_2_II	synthetic metagenome
SAMN29939632	Mock_F_2_III	synthetic metagenome

Supplementary Table 3. Illumina MiSeq BioSample accessions

IDs of MiSeq sequences stored in SRA database for identifying each sample (BioProject ID PRJNA862334)

Accession	Sample Name	Organism
SAMN29967857	A1_T1_R1	synthetic metagenome
SAMN29967858	A1_T1_R2	synthetic metagenome
SAMN29967859	A1_T2_R1	synthetic metagenome
SAMN29967860	A1_T2_R2	synthetic metagenome
SAMN29967861	A1_T3_R1	synthetic metagenome
SAMN29967862	A1_T3_R2	synthetic metagenome
SAMN29967863	A2_T1_R1	synthetic metagenome
SAMN29967864	A2_T1_R2	synthetic metagenome
SAMN29967865	A2_T2_R1	synthetic metagenome
SAMN29967866	A2_T2_R2	synthetic metagenome
SAMN29967867	A2_T3_R1	synthetic metagenome
SAMN29967868	A2_T3_R2	synthetic metagenome
SAMN29967869	B1_T1_R1	synthetic metagenome
SAMN29967870	B1_T1_R2	synthetic metagenome
SAMN29967871	B1_T2_R1	synthetic metagenome
SAMN29967872	B1_T2_R2	synthetic metagenome
SAMN29967873	B1_T3_R1	synthetic metagenome
SAMN29967874	B1_T3_R2	synthetic metagenome
SAMN29967875	B2_T1_R1	synthetic metagenome
SAMN29967876	B2_T1_R2	synthetic metagenome
SAMN29967877	B2_T2_R1	synthetic metagenome
SAMN29967878	B2_T2_R2	synthetic metagenome
SAMN29967879	B2_T3_R1	synthetic metagenome
SAMN29967880	B2_T3_R2	synthetic metagenome
SAMN29967881	C1_T1_R1	synthetic metagenome
SAMN29967882	C1_T1_R2	synthetic metagenome
SAMN29967883	C1_T2_R1	synthetic metagenome
SAMN29967884	C1_T2_R2	synthetic metagenome
SAMN29967885	C1_T3_R1	synthetic metagenome
SAMN29967886	C1_T3_R2	synthetic metagenome
SAMN29967887	C2_T1_R1	synthetic metagenome
SAMN29967888	C2_T1_R2	synthetic metagenome
SAMN29967889	C2_T2_R1	synthetic metagenome
SAMN29967890	C2_T2_R2	synthetic metagenome
SAMN29967891	C2_T3_R1	synthetic metagenome

SAMN29967892	C2_T3_R2	synthetic metagenome
SAMN29967893	D1_T1_R1	synthetic metagenome
SAMN29967894	D1_T1_R2	synthetic metagenome
SAMN29967895	D1_T2_R1	synthetic metagenome
SAMN29967896	D1_T2_R2	synthetic metagenome
SAMN29967897	D1_T3_R1	synthetic metagenome
SAMN29967898	D1_T3_R2	synthetic metagenome
SAMN29967899	D2_T1_R1	synthetic metagenome
SAMN29967900	D2_T1_R2	synthetic metagenome
SAMN29967901	D2_T2_R1	synthetic metagenome
SAMN29967902	D2_T2_R2	synthetic metagenome
SAMN29967903	D2_T3_R1	synthetic metagenome
SAMN29967904	D2_T3_R2	synthetic metagenome
SAMN29967905	E1_T1_R1	synthetic metagenome
SAMN29967906	E1_T1_R2	synthetic metagenome
SAMN29967907	E1_T2_R1	synthetic metagenome
SAMN29967908	E1_T2_R2	synthetic metagenome
SAMN29967909	E1_T3_R1	synthetic metagenome
SAMN29967910	E1_T3_R2	synthetic metagenome
SAMN29967911	E2_T1_R1	synthetic metagenome
SAMN29967912	E2_T1_R2	synthetic metagenome
SAMN29967913	E2_T2_R1	synthetic metagenome
SAMN29967914	E2_T2_R2	synthetic metagenome
SAMN29967915	E2_T3_R1	synthetic metagenome
SAMN29967916	E2_T3_R2	synthetic metagenome
SAMN29967917	F1_T1_R1	synthetic metagenome
SAMN29967918	F1_T1_R2	synthetic metagenome
SAMN29967919	F1_T2_R1	synthetic metagenome
SAMN29967920	F1_T2_R2	synthetic metagenome
SAMN29967921	F1_T3_R1	synthetic metagenome
SAMN29967922	F1_T3_R2	synthetic metagenome
SAMN29967923	F2_T1_R1	synthetic metagenome
SAMN29967924	F2_T1_R2	synthetic metagenome
SAMN29967925	F2_T2_R1	synthetic metagenome
SAMN29967926	F2_T2_R2	synthetic metagenome
SAMN29967927	F2_T3_R1	synthetic metagenome
SAMN29967928	F2_T3_R2	synthetic metagenome

Supplementary Table 4. Reference sequences length of organisms included in mock communities the most common ghosts

Species	Length of reference sequence in UNITE (bp)
Orbiliales_sp|UDB014954|SH1654757.08FU|	1390
Nakaseomyces_sp|MF767857|SH1505584.08FU|	1360
Glaciozyma_sp|LC493225|SH2711413.08FU|	1193
Saccharomyces_paradoxus|AJ229059|SH2466103.08FU|	766
Saccharomyces_bayanus|KJ707146|SH1613179.08FU|	758
Hanseniaspora_uvarum|AJ271031|SH1921544.08FU|	710
Debaryomyces_hansenii|AB053101|SH2154634.08FU|	576
Debaryomyces_udenii|JN942656|SH1516594.08FU|	550
Debaryomyces_robertsiae|AB054019|SH1676795.08FU|	576
Rhodotorula_mucilaginosa|KJ706479|SH1558748.08FU|	558
Rhodotorula_diobovata|MH753702|SH1558776.08FU|	539
Dipodascus_sp|MG776363|SH2708874.08FU|	294
Metschnikowia_pulcherrima|KY104205|SH2751425.08FU|	294
Metschnikowia_sp|KY197816|SH3341279.08FU|	479
Candida_haemulonis|AY500375|SH1555159.08FU|	285
